# Culture with apically applied healthy or disease sputum alters the airway surface liquid proteome and ion transport across human bronchial epithelial cells

**DOI:** 10.1152/ajpcell.00234.2021

**Published:** 2021-10-06

**Authors:** Maximillian Woodall, Boris Reidel, Mehmet Kesimer, Robert Tarran, Deborah L. Baines

**Affiliations:** ^1^Institute for Infection and Immunity, St George’s, University of London, Cranmer Terrace, Tooting, London, United Kingdom; ^2^Department of Pathology and Laboratory Medicine, University of North Carolina at Chapel Hill, Chapel Hill, North Carolina; ^3^Department of Cell Biology & Physiology, University of North Carolina at Chapel Hill, Chapel Hill, North Carolina

**Keywords:** CFTR, epithelial cell, epithelium, ion transport, proteomics, sputum

## Abstract

Airway secretions contain many signaling molecules and peptides/proteins that are not found in airway surface liquid (ASL) generated by normal human bronchial epithelial cells (NHBEs) in vitro. These play a key role in innate defense and mediate communication between the epithelium, the immune cells, and the external environment. We investigated how culture of NHBE with apically applied secretions from healthy or diseased (cystic fibrosis, CF) lungs affected epithelial function with a view to providing better in vitro models of the in vivo environment. NHBEs from 6 to 8 different donors were cultured at air-liquid interface (ALI), with apically applied sputum from normal healthy donors (normal lung sputum; NLS) or CF donors (CFS) for 2–4 h, 48 h, or with sputum reapplied over 48 h. Proteomics analysis was carried out on the sputa and on the NHBE ASL before and after culture with sputa. Transepithelial electrical resistance (TEER), short circuit current (I_sc_), and changes to ASL height were measured. There were 71 proteins common to both sputa but not ASL. The protease:protease inhibitor balance was increased in CFS compared with NLS and ASL. Culture of NHBE with sputa for 48 h identified additional factors not present in NLS, CFS, or ASL alone. Culture with either NLS or CFS for 48 h increased cystic fibrosis transmembrane regulator (CFTR) activity, calcium-activated chloride channel (CaCC) activity, and changed ASL height. These data indicate that culture with healthy or disease sputum changes the proteomic profile of ASL and ion transport properties of NHBE and this may increase physiological relevance when using in vitro airway models.

## INTRODUCTION

Airway secretions contain signaling molecules and peptides/proteins that play a key role in innate defense and mediate communication between the external environment, inflammatory cells, and the epithelium. Reciprocal activity of the cystic fibrosis transmembrane regulator (CFTR) anion channel and the epithelial Na^+^ channel (ENaC) is important for maintaining the correct hydration of the airway surface liquid (ASL), mucociliary clearance, and innate immune function of the airways. In respiratory diseases such as cystic fibrosis (CF), aberrant CFTR transport results in an altered luminal environment. Changes to mucus structure, ASL volume, pH, protease activity, pathogenic bacteria and their toxins, inflammatory cells, and inflammatory mediators all contribute to the development of a thick viscous sputum (1–5). These changes can then, in turn, further modify CFTR and ENaC activity. Normal human bronchial epithelial cells (NHBEs), cultured at air liquid interface in vitro, produce a similarly regulated ASL, but this lacks contribution from other cell types found in vivo. Exposure of NHBE to supernatant from mucopurulent material (SMM) collected from cadaver or posttransplant lungs was shown to increase CFTR activity and airway surface liquid (ASL) height (1, 6). Proinflammatory mediators present in SMM were proposed to be responsible for this effect (1, 6). In addition, induced sputum from people with CF (CFS) was shown to evoke an acute protease-mediated effect that activated ENaC, driving dehydration of the airways (5). These findings indicate that factors present in the disease sputum can have short-term and long-term effects on ion channel activity in vitro.

However, most of the observed effects of exposure to SMM or CFS in vitro have been compared to exposure with a buffered salt solution. Normal healthy lungs do not contain mucopurulent material but sputum can be induced and collected (normal lung sputum, NLS). The effect of NLS on epithelial function is poorly understood, but we proposed that culture of NHBE with apically applied NLS could provide an improved physiological control to determine epithelial changes mediated exclusively by the pathophysiological constituents of sputum associated with disease states such as CFS. In addition, exposure to apical factors such as sputum in vitro has often relied on only a single application for the duration of the treatment. In vivo, factors are continually secreted into the ASL from the epithelium and inflammatory cells. The mucociliary escalator has also been calculated to bring 50 µL of new ASL to the tracheaobronchial region every 24 h (7). Some of these factors may be subject to rapid degradation/inactivation reducing their activity. Accounting for the labile nature of proteins in vitro is challenging, nevertheless, we wanted to address this by reapplying sputum to airway cultures to replenish labile constituents of the sputum in a chronic exposure setting.

Based on previous studies (1, 5, 6, 8), we therefore tested the hypotheses that culture of NHBE in vitro with apically applied NLS would not change the function of NHBE and that any responses to normal or disease sputum would be dependent on duration of application in culture. We investigated the proteomic constituents of the NLS, CFS, and NHBE ASL before and after culture with apically applied sputa, short circuit current (I_sc_), and ASL height. We used a 2–4 h “acute” (5) and a 48 h “chronic” culture with apically applied sputa (1) using primary bronchial airway epithelial cells from 6 to 8 different donors without respiratory disease. We studied the effects of reapplication of apical sputa over 48 h to better replicate conditions in vivo. The aim was to understand how secretions from the normal luminal environment modify airway epithelial function to provide better in vitro models of airway function.

## MATERIALS AND METHODS

### Primary Normal Human Bronchial Epithelial Cell Culture

Normal human bronchial epithelial cells (NHBEs), from male and female, were obtained from endobronchial brushings or extracted from explanted nondiseased lungs as previously described and in accordance with ethical approvals obtained by The University of North Carolina at Chapel Hill Biomedical Institutional Review Board (Protocol No. 03-1396) (9). Donor demographics (NHBE) are shown in Supplemental Table S1a (all Supplemental material is available at https://doi.org/10.6084/m9.figshare.14791827). Cells were cultured on permeable supports and maintained at air-liquid interface (ALI) in a modified bronchial epithelial growth medium as described (9). Cells were studied 28–35 days after seeding on 12-mm T-clear inserts (Corning-Costar, Corning, NY).

**Table 1.** Transepithelial electrical resistance of NHBE from different donors

Donor	Mean, Ω·cm^2^	±SD	*n*
DD0190p1	1,169	36	6
DD028Np1	933	202	8
DD039Np1	845	47	10
DD064Np1	775	65	9
DD0130p1	533	39	10
DD0010p1	276	30	10

Donor number and passage number (p1) are shown with corresponding TEER (Ω^.^cm^2^) as means ± SD, for the number of individual cultures as shown (*n*). Donor demographics are given in Supplemental Table S1a. NHBE, normal human bronchial epithelial cell; TEER, transepithelial electrical resistance.

### Sputum Preparation

Airway sputum samples were obtained as previously described (10–12) and in accordance with ethical approvals obtained by The University of North Carolina at Chapel Hill Biomedical Institutional Review Board (Protocol No. 15-2431). Donor demographics (sputum) are provided in Supplemental Table S1b. An ultrasonic nebulizer was filled with 30 mL of 5% hypertonic saline (NaCl). After a 12-min inhalation period, the subjects underwent a cleansing procedure: gargle and rinse the mouth with water, scrape and clear the back of the throat (to avoid the inclusion of nonairway fluid samples), and blow nose. The subjects were asked to deliver a chesty cough and expectorate the secretions into a sterile specimen jar. Samples were placed on ice and stored at −80°C.

Unrefined sputum samples were thawed on ice and centrifuged at 4,000 *g* for 20 min to remove cells, bacteria, and macromolecules and the supernatant was used for all downstream experimentation. NLS or CFS was pooled from 10 donors and 20 μL was applied to the apical surface of ALI cultures. Cells were cultured with apically applied CFS or NLS for 2–4 h (acute) or 48 h (chronic), or to emulate the replacement of labile constituents in the luminal lung environment, 4 μL of NLS or CFS was reapplied apically twice a day for 48 h and 4 h before taking the first measurement (reapplied).

### Mass Spectrometry-Based Proteomic Analysis

NHBE ASL was acquired for mass spectrometry by incubating NHBE cultures at 37°C with 100-µL apical applied phosphate-buffered saline (PBS) for 10 min and collecting the resulting apical fluid. Samples were prepared for liquid chromatography tandem mass spectrometry (LC-MS/MS) analysis using filter-aided sample preparation (FASP) (13). Peptides for peptidomics analysis were collected using Amicon Ultra 4 10-kDa filters before the proteomic sample preparation. Samples were reduced by 10 mM dithiothreitol (Sigma-Aldrich) and alkylated in 50 mM iodoacetamide (Sigma-Aldrich), followed by digestion overnight using trypsin (20 ng/μL) in 50 mM ammonium bicarbonate at 37°C. Peptide eluates were vacuum freeze-dried and dissolved in 25 μL of 1% acetonitrile and 0.1% trifluoroacetic acid. Solubilized peptide material (5 μL) was loaded into a trap column for proteomic analysis in a Q Exactive mass spectrometer coupled to an UltiMate 3000 nano HPLC system, and data acquisition was performed as described (14).

### Proteomic Data Analysis

The raw data were processed and searched against the Uniprot protein database (*Homo sapiens*, April 2019) using the Proteome Discoverer 1.4 (Thermo Fisher Scientific) software. Parameters used in the Sequest search engine were 10-ppm mass accuracy for parent ions and 0.02 Da accuracy for fragment ions, two missed cleavages allowed. Carbamidomethyl modification for cysteines was set to fixed and methionine oxidation to variable. Scaffold 4.7.5 (Proteome Software Inc.) was used to validate MS/MS-based peptide and protein identifications. Peptide identifications were accepted if they could be established at greater than 95.0% probability by the Scaffold Local FDR algorithm. Protein identifications were accepted if they could be established at greater than 99.0% probability and contained at least two identified peptides. Protein probabilities were assigned by the Protein Prophet algorithm. Proteins that contained similar peptides and could not be differentiated based on MS/MS analysis alone were grouped to satisfy the principles of parsimony. The heat map and Euclidean hierarchical clustering were generated from Log10 mean ion current data obtained from samples and normalized to a common protein found at similar levels in all samples (TXN) using Morpheus at software.broadinstitute.org.

### Electrophysiological Measurements

Transepithelial ion transport was measured using the Ussing technique as previously described (15). Cultures were mounted onto sliders and inserted into EasyMount Ussing Chamber System. Apical and basolateral chambers were filled with 5 mL of buffer [117 mM NaCl, 2.5 mM CaCl_2_, 4.7 mM KCl, 1.2 mM MgSO_4_, 25 mM NaHCO_3_, 1.2 mM KH_2_PO_4_, 11 mM D-glucose, 5 mM HEPES (pH 7.4)]. The solution was maintained at 37°C and bubbled with 21% O_2_ + 5% CO_2_ premixed gas throughout the course of the experiment. The epithelium was voltage clamped at 0 mV, and short-circuit current (I_sc_) and transepithelial electrical resistance (TEER) were measured. Cultures were equilibrated for a minimum of 20 min before the addition of amiloride, 100 μM (apical) to inhibit ENaC; forskolin, 10 μM (bilateral) to elevate cyclic adenosine monophosphate (cAMP) and activate CFTR; CFTR_inh_172, 10 μM (apical) to inhibit CFTR; uridine triphosphate (UTP), 100 μM (apical) to activate Ca^2+^-activated Cl^−^ channels (CaCC) and ouabain, 100 μM.

### Airway Surface Liquid Height Measurements

PBS, CFS, or NLS (20 μL) containing 0.5 mg/mL of 10-kDa dextran-tetramethylrhodamine (Life Technologies) was added to the apical surface of NHBE. Cells were labeled with CellTrace Calcein Green, AM (Thermo Fisher; 5 µg/mL in media). ASL height was stabilized by 120 min. Vasoactive intestinal peptide (100 nM) (VIP; Life Technologies) was added basolaterally to induce CFTR-mediated secretion. Perfluorocarbon (3 M Fluorinert FC-770) was added apically to prevent ASL evaporation. Images were obtained immediately before and 60 min after basolateral VIP addition in XZ-scanning mode by using a Leica SP8 confocal microscope with a ×63/1.3 numerical aperture (NA) glycerol immersion lens. Ten ASL images per culture were acquired by using an automatic stage with the “Mark-and-Find” function as described (16).

### Statistics

Raw data from cells from individual donors before and after treatment (as shown in supplementary data) was analyzed using a nonparametric paired Wilcoxon test. Normalized summary data shown in the manuscript was analyzed using a nonparametric Kruskal–Wallis test with a post hoc Dunn’s test. Data are shown as individual points (scatterplot) with means ± standard deviation. Significant differences are indicated with *P < 0.05, **P < 0.01, and ***P < 0.001. Data analyses were performed using GraphPad Prism 7.0 (GraphPad Software, La Jolla, CA).

## RESULTS

### Sputum/ASL Proteomes

**Figure 1.**
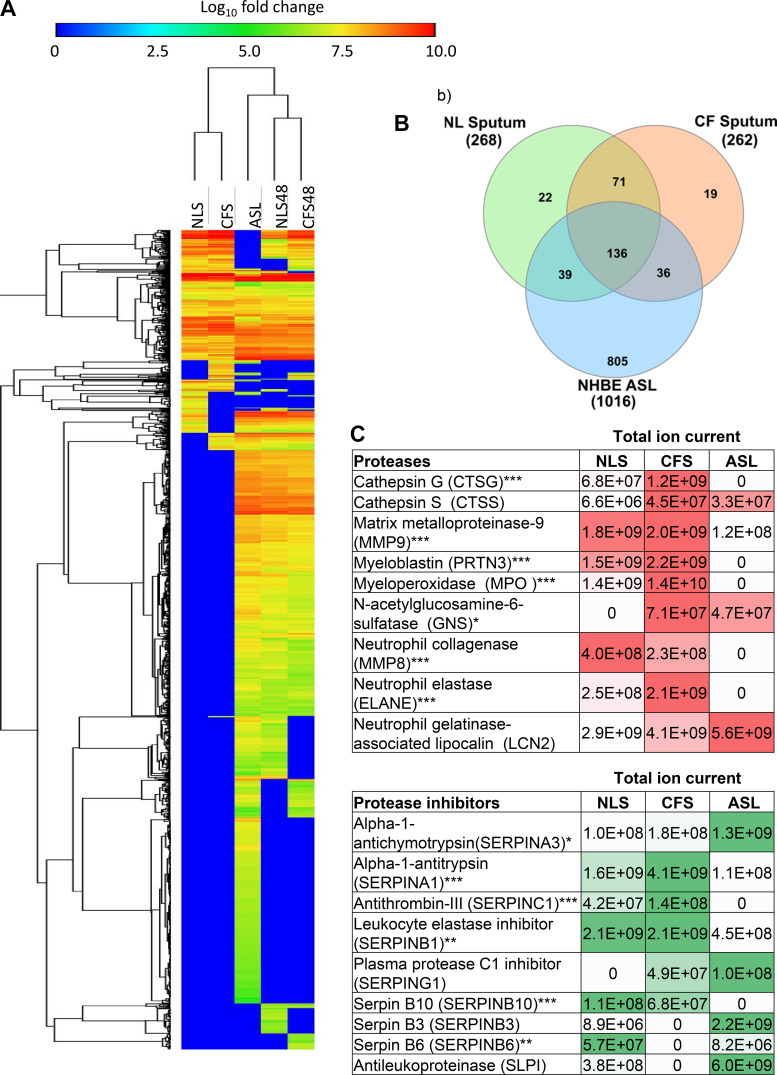
Distribution and relative abundance of proteins detected in samples of CFS, NLS, and ASL from NHBE before and after exposure to sputa. *A*: heat map with Euclidean distance hierarchical clustering showing distribution and relative abundance of proteins identified in NLS, CFS (pooled sputum samples) (*n* = 10), and ASL before culture with sputum (ASL) (*n* = 8) and after culture with apically applied NLS (NLS48) or CFS (CFS48) for 48 h (both *n* = 4). *B*: Venn diagram showing number of unique proteins found in NLS, CFS, or NHBE ASL. *C*: the identity and abundance of proteases and protease inhibitors related to CF lung disease pathology in NLS and ASL before exposure to sputum. Total ion current (TIC) is relative to the abundance of proteins within a 5-µL sample, for each row intensity of shading (red for proteases; green for protease inhibitors) highlight the highest value. ASL, airway surface liquid; CF, cystic fibrosis; CFS, cystic fibrosis sputum; NHBE, normal human bronchial epithelial cell; NLS, normal healthy donor. Significantly different between groups *P < 0.05, **P < 0.01, ***P < 0.001.

We first analyzed the proteomes of the NLS and CFS (pooled samples *n* = 10). We then analyzed NHBE ASL (pooled *n* = 8) before and after culture with sputum (pooled *n* = 4 each). A total of 268 proteins were identified in NLS, 262 in CFS and 1,016 in NHBE ASL (Fig. 1, *A* and *B*). The variation in the number of proteins identified is likely due to differences in protein concentration associated with the different methods of sample acquisition. The signal intensity for MUC5B in the NLS analysis was approximately three times lower than for a NHBE ASL samples (Supplemental Table S2). Thus, there was an increase in sensitivity for acquiring proteins in ASL over that of induced sputum. One hundred and thirty-six proteins were common to all samples, whereas 22 proteins were exclusively found in NLS, 19 in CFS, and 805 in NHBE ASL (Fig. 1*B*). The proteins exclusively found in CFS were tightly correlated with immunity pathways including regulated exocytosis, neutrophil degranulation, immune response, cell activation, and transport (alignments made via analysis through the STRING database; *P* < 0.001). No such correlations were identified with proteins exclusively found in NLS. Both NLS and NHBE ASL contained a lower abundance of identified proteases compared with CFS, in particular, neutrophil elastase, cathepsins, myeloperoxidase, and myeloblastin (Fig. 1*C*). NLS contained more serpin family protein members than CFS and NHBE ASL, although key protease inhibitors such as α-1-antitrypsin, antithrombin, and leukocyte elastase inhibitor were elevated in CFS (Fig. 1*C*). Innate immune factors such as Neutrophil defensin 3, bactericidal permeability increasing fold-containing family B member 2 (BPIFB2), and Lipocalin-1 were detected in both NLS and CFS. Short palate, lung, and nasal epithelial clone 1 (or BPIFA1) (SPLUNC1), reported inhibitor of ENaC and a potential therapeutic peptide for CF, was detected in NHBE ASL (Supplemental Table S2).

**Figure 2.**
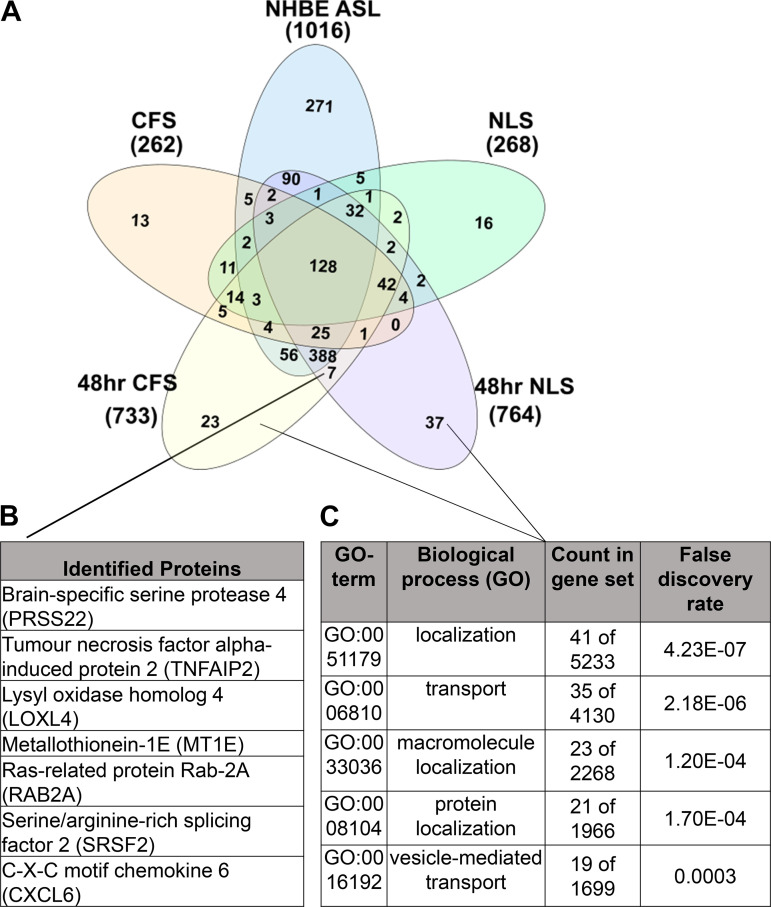
The number, identity, and relative abundance of key proteins detected in ASL from NHBE chronically cultured with apically applied CFS or NLS. *A*: Venn diagram showing unique proteins identified in NLS, CFS (pooled, *n* = 10), ASL before (NHBE ASL) (*n* = 8) or after 48 h culture with NLS (48 h NLS) (*n* = 4) or CFS (48 h CFS) (*n* = 4). *B:* the seven proteins exclusive to both chronically exposed NLS and CFS are shown. *C*: the 60 proteins found exclusively in chronically cultured NHBE were aligned to biological processes through the STRING database, the top five matches are shown alongside their false discovery rate (*P* < 0.001). ASL, airway surface liquid; CFS, cystic fibrosis sputum; NHBE, normal human bronchial epithelial cell; NLS, normal healthy donor.

Analysis of the ASL from NHBE cultured with NLS or CFS identified seven new proteins resulting from chronic exposure to either sputa (Fig. 2, *A* and *B*), which included brain-specific protease 4 (*PRSS22*), a serine protease found in airway cells, lung, and esophagus, and the tumor necrosis factor alpha (TNF-α)-induced protein 2 (*TNFAIP2*), which mediates many of the effects of TNF-α. There were 37 proteins exclusively identified in NLS-exposed ASL and 23 in CFS-exposed ASL, indicating that these proteins were released from NHBE in response to sputum exposure. Evidence indicates that brain-specific protease 4 is constitutively expressed in airway epithelium, is resistant to protease inhibitors such as a1-anti-trypsin and that urokinase-type plasminogen activator is a preferred substrate, suggesting that brain-specific protease 4 could have a role in lung injury and repair (17, 18). TNF-α-induced protein 2 is a factor in the early response to inflammatory stress. The other 37 proteins were aligned to biological processes in the STRING database and were found to be tightly correlated with cellular transport and localization processes, including protein localization and vesicle-mediated transport (*P* < 0.001) (Fig. 2, *A* and *C*). This is a well-documented response to inflammatory signaling in eukaryotic cells, in which transcriptional induction of genes that have functions associated with increasing the endoplasmic reticulum (ER) volume and capacity for protein folding are upregulated (19–21). A number of factors in sputum (including proteases) could elicit the release of these proteins via direct or receptor-mediated mechanisms. More work is required to understand how/why sputum induces release of these proteins from airway cells in vitro.

### Effect of NLS and CFS on Epithelial Electrophysiology

**Figure 3.**
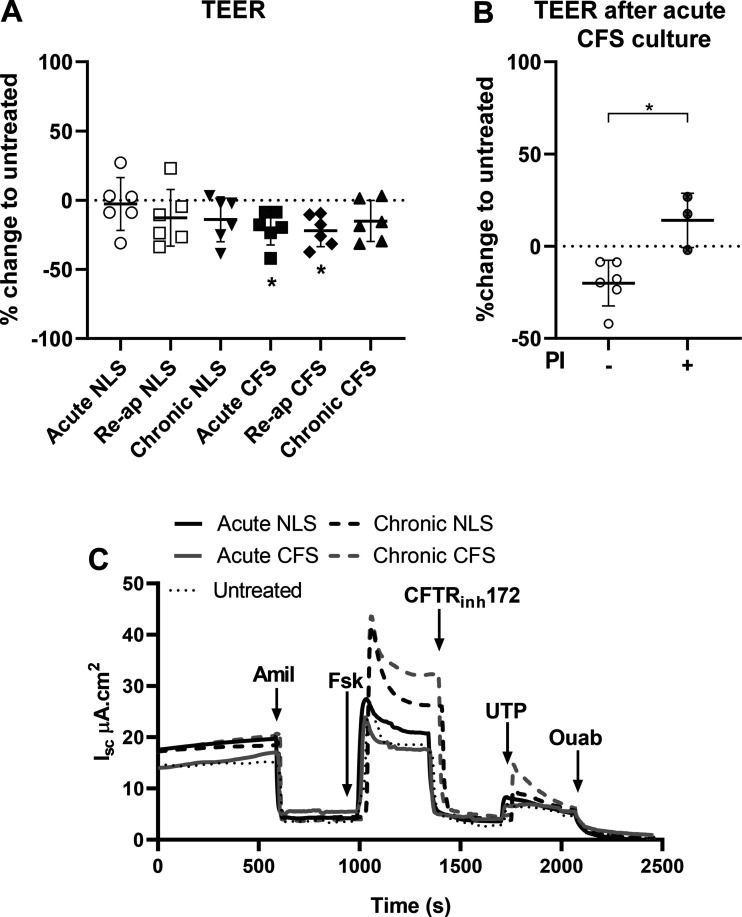
Culture with apically applied CFS but not NLS decreased NHBE TEER, but culture with either sputa changed short circuit current (I_sc_). *A*: percent change in TEER of NHBE before and after culture with NLS or CFS for 4 h (acute) or 48 h (chronic) or reapplication of sputa over 48 h (Re-ap) or CFS incubated with protease inhibitor (acute CFS+PI), normalized to change in untreated cultures over the same time period. Presented as individual points, means ± SD, for *n* = 6 different donors. *Statistically different from untreated control, *P* < 0.05. *B*: percent change in TEER of NHBE before and after culture with CFS for 4 h with (+) and without (−) cOmplete protease inhibitor cocktail. *Statistically different *P* < 0.05. *C*: exemplar I_sc_ traces from NHBE from one donor that were untreated (dotted line) or after acute (4 h: solid lines) or chronic (48 h: dashed lines) exposure to NLS or CFS, showing addition of specific activators and inhibitors of ion transport (marked by black arrows); amiloride (100 μM), forskolin (10 μM), CFTR inhibitor 172 (CFTRinh, 10 μM), UTP (100 μM), and ouabain (100 μM). All drugs were added apically with exception of forskolin, which was also added basolaterally. CFS, cystic fibrosis sputum; CFTR cystic fibrosis transmembrane regulator; NHBE, normal human bronchial epithelial cell; NLS, normal healthy donor; TEER, transepithelial electrical resistance; UTP, uridine triphosphate.

Mean donor resistance ranged from 276 Ω.cm^2^ to 1,169 Ω.cm^2^ but with little variation within samples from the same donor (Table 1, Supplemental Table S1a). TEER changed in untreated NHBE at both the acute and chronic time points (Supplemental Fig. S1, *a–h*). Taking these changes into account, NLS had no further effect on TEER. The only consistent response we observed was a reduction in TEER (∼20%) after acute and reapplied culture with CFS compared with untreated NHBE (*P* < 0.05; *n* = 6, respectively; Fig. 3*A*). Incubation of CFS for 30 min with cOmplete protease inhibitor cocktail prevented the decrease in TEER (*P* < 0.01, *n* = 4; Fig. 3*B*). These data indicated that proteases present in CFS acutely reduce epithelial TEER.

There was significant variation between basal short circuit current (I_sc_) recorded for each donor, and there were donor-dependent differences in the magnitude of the response to pharmacological drugs when different sputa treatments were applied. Nevertheless, specific sputum exposures resulted in consistent responses in all donors as exemplified by donor DD0028N (Fig. 3*C*).

### Effect of NLS

**Figure 4.**
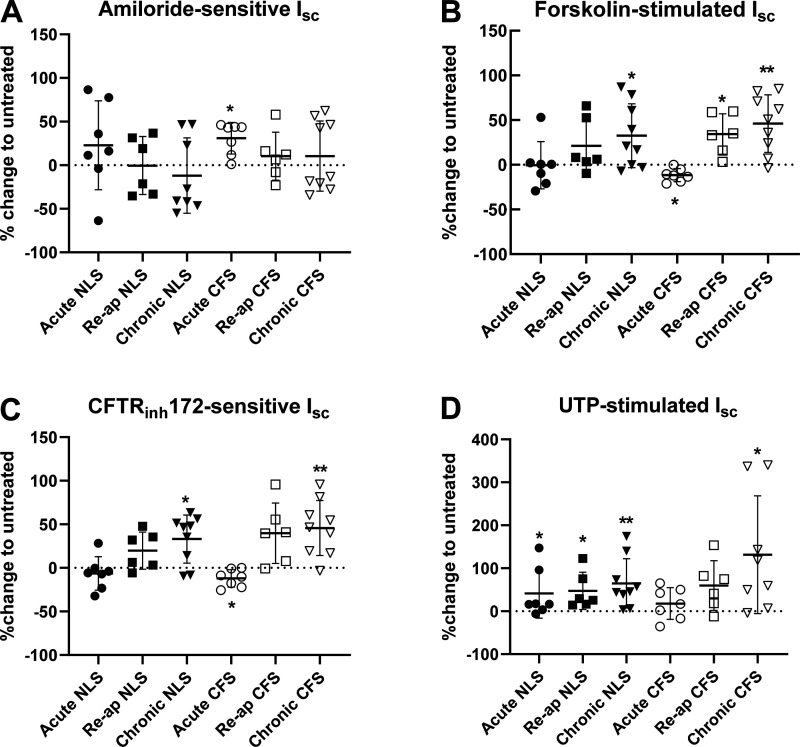
Acute or chronic culture of NHBE with sputa induced different effects on I_sc_ in multiple donors. I_sc_ across NHBE before (untreated) and after culture with NLS or CFS for 4 h (acute) or 48 h (chronic) or reapplication of sputa over 48 h (Re-ap) for *n* = 6–8 individual donors. The dotted line at zero represented no change, positive variation indicates increased I_sc_, and negative variation indicates decreased I_sc_ in response to treatment. Amiloride-sensitive I_sc_ (*A*), forskolin-stimulated I_sc_ (*B*), CFTR_inh_172 –sensitive I_sc_ (*C*), UTP-stimulated I_sc_ (*D*). Mean values are shown as horizontal bars ± SD. Significantly different from untreated control *P < 0.05; **P < 0.01. CFS, cystic fibrosis sputum; CFTR_inh_ : cystic fibrosis transmembrane regulator inhibitor; I_sc_: short circuit current; NHBE, normal human bronchial epithelial cell; NLS, normal healthy donor; UTP, uridine triphosphate.

Acute culture with apically applied NLS increased UTP-sensitive I_sc_ (*P* < 0.05, *n* = 7), but had no consistent effect on any other parameter tested (Fig. 4*D*). Chronic culture with NLS, however, resulted in an increase in both forskolin-stimulated, CFTR_inh_172-sensitive (*P* < 0.05, *n* = 8), and UTP-stimulated I_sc_ (*P* < 0.01, *n* = 8; Fig. 4, *BD*). Reapplying NLS increased UTP-stimulated I_sc_ (*P* < 0.05, *n* = 6; Fig. 4*D*). However, we observed that the changes in I_sc_ were generally lower than those elicited by chronic application of NLS. Thus, reapplying NLS tempered the effects of treatment on CFTR and CaCC activity. The responses to culture with/without NLS by individual donors are shown in Supplemental Fig. S2, *ai*.

### Effect of CFS

Consistent with previous findings, acute culture with apically applied CFS increased the amiloride-sensitive I_sc_ in each donor (*P* < 0.05; *n* = 7; Fig. 4*A*). It also elicited a modest decrease in forskolin-stimulated I_sc_ and CFTR_inh_172-sensitive I_sc_ compared with the untreated samples (*P* < 0.05; *n* = 7, respectively) (Fig. 4, *B* and *C*). Thus, acute exposure to CFS increased ENaC and decreased CFTR activity.

Chronic culture with apically applied CFS increased forskolin-stimulated, CFTR_inh_172-sensitive, and UTP-stimulated I_sc_ (*P* < 0.05, *n* = 8, respectively; Fig. 4, *B–D*). These data indicate that chronic exposure to CFS increased both CFTR and CaCC activity. When CFS was reapplied over a period of 48 h, the predominant effect determined was that forskolin-stimulated I_sc_ remained elevated (*P* < 0.05, *n* = 6; Fig. 4*B*). But again, we observed that the changes in I_sc_ were generally lower than those elicited by chronic culture with CFS. Thus, reapplying CFS tempered the chronic effects of CFTR and CaCC activity. The responses to culture with/without CFS by individual donors are shown in Supplemental Fig. S3, *ai.*

### Effect of NLS and CFS on ASL Height

**Figure 5.**
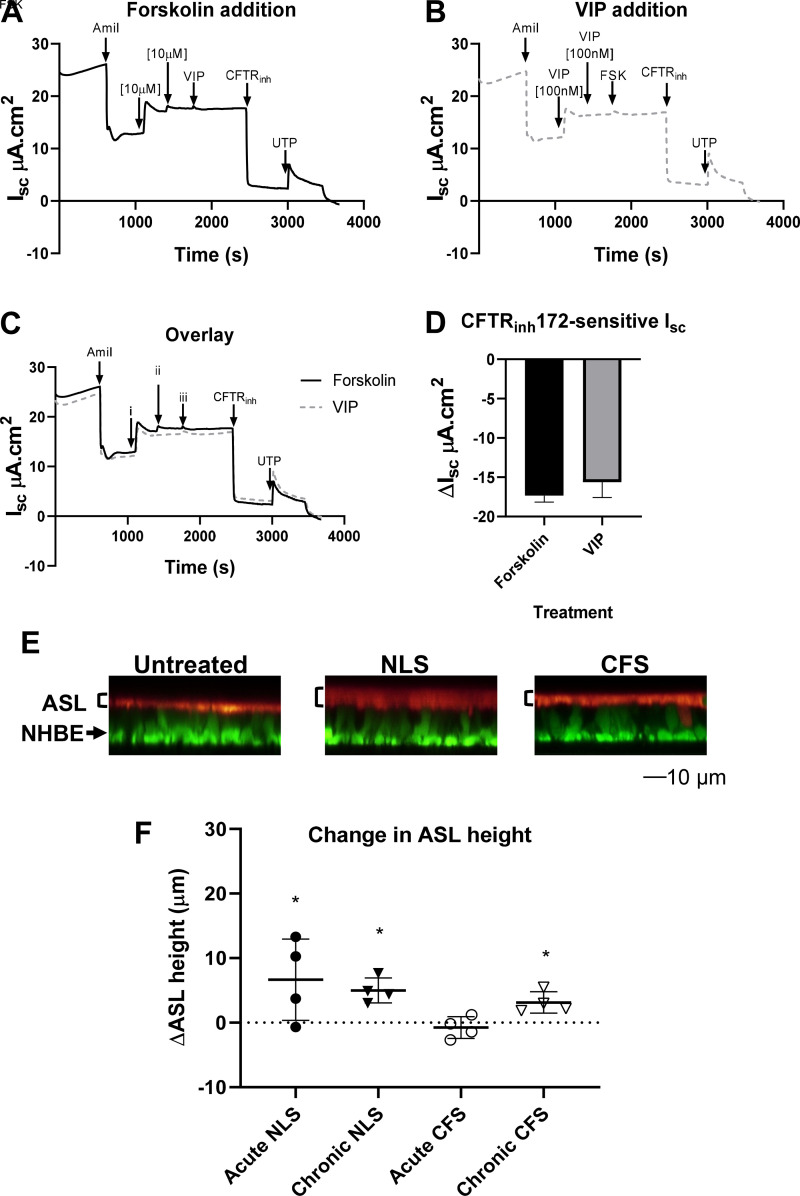
ASL height is modified by culture with CFS and NLS. Example I_sc_ traces from NHBE with: forskolin addition prior to vasoactive intestinal peptide (VIP) addition (*A*), VIP addition prior to forskolin (FSK) (*B*), and overlay (*C*). Overlay of *A* and *B* with addition of specific activators and inhibitors (i, ii and iii) as shown in *A* and *B* at points indicated by arrows. *D*: summary of bioelectric data for NHBE showing ΔI_sc_ in response to CFTR_inh_172 (CFTR_inh_, 10 μM) (*n* = 3). All drugs were added apically with the exception of forskolin, which was also added basolaterally. *E*: representative XZ images from one donor of ASL labeled with dextran-tetramethylrhodamine (red layer as indicated to right of image) overlying NHBE labeled with CellTrace Calcein Green, AM (green) following acute culture with CFS, NLS, or PBS (untreated). *F*: change in ASL height (µm) 1 h after basolateral addition of vasoactive intestinal peptide (VIP) after CFS and NLS culture for 4 h (acute) or 48 h (chronic). The dotted line represents no change after VIP treatment. Positive deflections from line represent increased ASL height after VIP treatment. Data are presented as individual points representing donor average values with mean values shown as horizontal bars ± SD (*n* = 2–4 from 4 to 7 different donors, untreated not shown). *Significantly different from untreated control *P* < 0.05. ASL, airway surface liquid; CFS, cystic fibrosis sputum; CFTR_inh_: cystic fibrosis transmembrane regulator inhibitor; I_sc_: short circuit current; NHBE, normal human bronchial epithelial cell; NLS, normal healthy donor; PBS, phosphate-buffered saline.

Vasoactive intestinal peptide (VIP) acts as an agonist of basolateral VPAC1 receptors, which subsequently increases intracellular cAMP concentration to increase CFTR activity (similar to the mechanisms of forskolin; 22). As VIP requires basolateral application only, it allowed us to better control apical fluid volume and the measurement of ASL height. In our model, the concentration of VIP used produced similar changes in I_sc_ to that of forskolin (Fig. 5, *A–D*). Treatment with VIP increased ASL height in NHBE acutely cultured with NLS but not acutely cultured with CFS (*P* < 0.05; *n* = 4; Fig. 5, *E* and *F*). VIP also increased ASL height after chronic culture with NLS or CFS (*P* < 0.05; *n* = 4; Fig. 5, *E* and *F*) consistent with increased Cl^−^-driven fluid secretion. VIP did not increase ASL height after acute culture with CFS, which correlated well with reduced Cl^−^ secretion and increased Na^+^-driven fluid absorption. Cultures with reapplied NLS and CFS were unsuitable for ASL height imaging, as 4-µL additions were insufficient in volume to produce homogenous dispersal of labeled dextran across the NHBE culture.

## DISCUSSION

Our data support and expand on findings that the complex luminal environment of the lung modifies airway epithelial function. Our demonstration that normal lung sputum changes NHBE function raises questions as to whether the current in vitro culture methods provide an appropriate model of in vivo ASL. In this study, we compared the proteomic composition of induced sputum from healthy individuals (NLS) to that from people with CF (CFS) and tested their effects on NHBE isolated from 6 to 8 different donors. There was significant variability in the response to culture with apically applied sputa associated with different donors and surprisingly, neither the TEER nor basal I_sc_ was predictive of the outcome. Nevertheless, some treatments generated similar response in all donors and, importantly, we were able to determine consistent acute and chronic effects of culture with both NLS and CFS on epithelial function lending power to our study.

We used proteome analysis to characterize the sputa and identify changes in the NHBE ASL in response to chronic culture with sputa. The data we obtained indicated that NLS, CFS, and ASL had distinct proteomic fingerprints. Consistent with the findings of others, CFS contained more immunity-related proteins and inflammation-related proteases than NLS (23–25). NLS contained a broader range of protease inhibitors supporting the notion that the protease/protease inhibitor balance is increased in CF disease (1, 3, 5, 26). Interestingly, although NHBE ASL showed commonality with both NLS and CFS, the protease/protease inhibitor balance was more akin to that of NLS.

Consistent with the proteomic data, culture of NHBE with CFS for 2–4 h reduced TEER in a protease-dependent manner. Proteases have been shown to significantly increase transepithelial diffusion, decrease tight junction protein formation, and reduce epithelial apical junctional complex reformation during wound healing in vitro (2, 27, 28). CFS but not NLS also increased ENaC activity, reduced CFTR activity, and ASL height as previously reported (5). This finding was interesting in the face of a reduction of TEER, which might be expected to increase fluid flux into the ASL, but the acute response to CFS mimicked CF disease where ASL height is reduced in vivo (29, 30). Protease activity was also likely to underpin the increase in ENaC and decrease in CFTR activity. For example, neutrophil elastase (which we found to be ∼10-fold more abundant in CFS than in NLS) has been shown to rapidly increase ENaC activity in human nasal epithelial cells and decrease CFTR channel function within 6 h in NHBE and mice in vivo (31, 32). Cathepsins, serine proteases, and matrix metalloproteinases, which we show are elevated in CFS [corroborating with other work (2, 3, 33, 34)], are known to cleave and activate ENaC (35–37). In addition, the inhibitor of ENaC, SPLUNC1, although present in NHBE ASL was not detected in CFS or CFS-treated cultures where it is reportedly degraded (5).

We showed that chronic culture with apically applied sputa evoked changes to the proteome of the NHBE ASL that were distinct from NLS and CFS and signposted an epithelial response to the apical application of the sputum that further changed the luminal environment. Disproving our hypothesis, we also found that chronic culture with either NLS or CFS consistently increased anion transport via CFTR and CaCC activity in all donor NHBE, and the increased capacity for Cl^−^ efflux correlated with increased fluid secretion into the ASL. Chronic exposure to supernatant from mucopurulent material (SMM) was shown to increase CFTR and CaCC activity (1, 6). Although we cannot exclude indirect effects that modify the driving force for Cl^−^ movement, our findings indicate that chronic culture with sputum from healthy donors or patients with CF with FEV1s ≥ 0.92 (i.e., cohorts still responsive to treatments and not requiring lung transplant) had a similar effect to that of SMM on electrophysiological readouts. This novel finding raises the possibility that factors present in both sputa and/or induced/released by epithelial cells in response to apical culture with sputa were responsible for the changes we observed.

Of the candidate proteins common to both CFS and NLS and in addition to those already described, olfactomedin-4 was present. Its role in the airway is not well understood, but it is reported to inhibit Cathepsin C-mediated protease activity [24; such as activation of immune cell-associated serine proteases (38, 39)] and play a role in epithelial differentiation (40), both of which are known to affect epithelial ion transport (31, 32, 35, 36, 41). Proinflammatory factors that were identified or that increased in response to exposure to sputa included TNFα-induced protein 2 (*TNFAIP2*) and interleukin 6 (IL-6). There were also a number of proteins associated with macromolecule localization, protein localization, and vesicle-mediated transport. Others have proposed inflammatory-mediated increases in CFTR expression via transcriptional and posttranscriptional mechanisms (1, 6, 8, 42). The CaCC, *TMEM16A* is also reported to be upregulated during inflammation and by associated endoplasmic reticulum Ca^2+^ store expansion (8, 42). Proteomic studies do not effectively identify proinflammatory cytokines or provide information on metabolites (24, 25, 43), and so, the specific mechanisms upregulating Cl^−^ efflux via CFTR and CaCC need further investigation. Furthermore, the net change of proteases present in the extracellular environment may affect protease-activated receptors, which are linked to a plethora of epithelial functions, including ion transport (44, 45). Nevertheless, we speculate that long-term culture with sputa per se to NHBE induces a proinflammatory/stress response that modifies CFTR and CaCC function. Finally, reapplying NLS or CSF resulted in a midway response in CFTR and CaCC activity. This implies a balance between factors involved in the acute versus chronic effects and questions the dominance of such factors in vivo.

In conclusion, our evidence indicates that culture of epithelial cells from different donors with healthy human sputum or sputum from people with CF disease changes the proteomic signature of the ASL. We show that there was a consistent response to chronic culture with apically applied sputa that is independent of a disease-specific effect. Our data support that acute acting components in sputum (that may be replenished in vivo but not in vitro) can modify outcomes. As a corollary to these novel findings, because the response to NLS was different to that reported for PBS (1, 6), we propose that culture of airway epithelial cells with NLS provides a more physiologically relevant control for the luminal environment and might provide a new method for functional studies investigating interventions/therapeutics for respiratory disease.

## SUPPLEMENTAL DATA

Supplemental Tables S1–S2 and Supplemental Figs. S1–S3: https://doi.org/10.6084/m9.figshare.14791827.

## GRANTS

Funded by the Cystic Fibrosis Trust Project No. SRC 006, Personalized Engineered Cell Therapies for Cystic Fibrosis. Travel cost for visits to UNC, Chapel Hill, were supported by Physiological Society Travel Grant and St. George’s University, Infection and Immunity Staff Development Fund. Provision of cells and media was supported by TARRAN17GO and BOUCHE15RO from the Cystic Fibrosis Foundation, and P30 DK065988 from the NIH.

## DISCLOSURES

No conflicts of interest, financial or otherwise, are declared by the authors.

## AUTHOR CONTRIBUTIONS

D.L.B. and R.T. conceived and designed research; M.W., B.R., and M.K. performed experiments; M.W., B.R., and M.K. analyzed data; M.W., B.R., M.K., and R.T. interpreted results of experiments; M.W. and M.K. prepared figures; M.W. drafted manuscript; D.L.B. edited and revised manuscript; M.W., B.R., M.K., R.T., and D.L.B. approved final version of manuscript.

## References

[1] Abdullah LH, Coakley R, Webster MJ, Zhu Y, Tarran R, Radicioni G, Kesimer M, Boucher RC, Davis CW, Ribeiro CMP. Mucin production and hydration responses to mucopurulent materials in normal versus cystic fibrosis airway epithelia. Am J Respir Crit Care Med 197: 481–491, 2018. doi:10.1164/rccm.201706-1139OC. 29099608 PMC5821906

[2] Gaggar A, Li Y, Weathington N, Winkler M, Kong M, Jackson P, Blalock JE, Clancy JP. Matrix metalloprotease-9 dysregulation in lower airway secretions of cystic fibrosis patients. Am J Physiol Lung Cell Mol Physiol 293: L96–L104, 2007. doi:10.1152/ajplung.00492.2006. 17384080

[3] Müller U, Hentschel J, Janhsen WK, Hünniger K, Hipler U-C, Sonnemann J, Pfister W, Böer K, Lehmann T, Mainz JG. Changes of proteases, antiproteases, and pathogens in cystic fibrosis patients’ upper and lower airways after IV-antibiotic therapy. Mediators Inflamm 2015: 626530, 2015., doi:10.1155/2015/626530. 26185365 PMC4491395

[4] Prulière-Escabasse V, Fanen P, Dazy AC, Lechapt-Zalcman E, Rideau D, Edelman A, Escudier E, Coste A. TGF-β1 downregulates CFTR expression and function in nasal polyps of non-CF patients. Am J Physiol Lung Cell Mol Physiol 288: L77–L83, 2005. doi:10.1152/ajplung.00048.2004. 15361357

[5] Webster MJ, Reidel B, Tan CD, Ghosh A, Alexis NE, Donaldson SH, Kesimer M, Ribeiro CMP, Tarran R. SPLUNC1 degradation by the cystic fibrosis mucosal environment drives airway surface liquid dehydration. Eur Respir J 52: 1800668, 2018. doi:10.1183/13993003.00668-2018. 30190268 PMC6547379

[6] Gentzsch M, Cholon DM, Quinney NL, Boyles SE, Martino MEB, Ribeiro CMP. The cystic fibrosis airway milieu enhances rescue of F508del in a pre-clinical model. Eur Respir J 52: 1801133, 2018. doi:10.1183/13993003.01133-2018. 30287473 PMC6482470

[7] Matsui H, Randell SH, Peretti SW, Davis CW, Boucher RC. Coordinated clearance of periciliary liquid and mucus from airway surfaces. J Clin Invest 102: 1125–1131, 1998. doi:10.1172/JCI2687. 9739046 PMC509095

[8] Pedrosa Ribeiro CM, Paradiso AM, Carew MA, Shears SB, Boucher RC. Cystic fibrosis airway epithelial Ca^2+^i signaling: the mechanism for the larger agonist-mediated Ca^2+^i signals in human cystic fibrosis airway epithelia. J Biol Chem 280: 10202–10209, 2005. doi:10.1074/jbc.M410617200. 15647273

[9] Fulcher ML, Randell SH. Human nasal and tracheo-bronchial respiratory epithelial cell culture. Methods Mol Biol 945: 109–121, 2013. doi:10.1007/978-1-62703-125-7_8. 23097104

[10] Alexis NE, Hu SC, Zeman K, Alter T, Bennett WD. Induced sputum derives from the central airways: confirmation using a radiolabeled aerosol bolus delivery technique. Am J Respir Crit Care Med 164: 1964–1970, 2001. doi:10.1164/ajrccm.164.10.2104051. 11734453

[11] Pin I, Freitag AP, O'Byrne PM, Girgis-Gabardo A, Watson RM, Dolovich J, Denburg JA, Hargreave FE. Changes in the cellular profile of induced sputum after allergen-induced asthmatic responses. Am Rev Respir Dis 145: 1265–1269, 1992. doi:10.1164/ajrccm/145.6.1265. 1595989

[12] Pin I, Gibson PG, Kolendowicz R, Girgis-Gabardo A, Denburg JA, Hargreave FE, Dolovich J. Use of induced sputum cell counts to investigate airway inflammation in asthma. Thorax 47: 25–29 1992. doi:10.1136/thx.47.1.25. 1539140 PMC463545

[13] Wiśniewski JR, Zougman A, Nagaraj N, Mann M. Universal sample preparation method for proteome analysis. Nat Methods 6: 359–362, 2009. doi:10.1038/nmeth.1322. 19377485

[14] Kesimer M, Cullen J, Cao R, Radicioni G, Mathews KG, Seiler G, Gookin JL. Excess secretion of gel-forming mucins and associated innate defense proteins with defective mucin un-packaging underpin gallbladder mucocele formation in dogs. PLoS One 10: e0138988, 2015. doi:10.1371/journal.pone.0138988. 26414376 PMC4586375

[15] Woollhead AM, Sivagnanasundaram J, Kalsi KK, Pucovsky V, Pellatt LJ, Scott JW, Mustard KJ, Hardie DG, Baines DL. Pharmacological activators of AMP-activated protein kinase have different effects on Na^+^ transport processes across human lung epithelial cells. Br J Pharmacol 151: 1204–1215, 2007. doi:10.1038/sj.bjp.0707343. 17603555 PMC2189835

[16] Choi HC, Kim CSK, Tarran R. Automated acquisition and analysis of airway surface liquid height by confocal microscopy. Am J Physiol Lung Cell Mol Physiol 309: L109–L118, 2015. doi:10.1152/ajplung.00027.2015. 26001773 PMC4504972

[17] Shetty S, Bhandary YP, Shetty SK, Velusamy T, Shetty P, Bdeir K, Gyetko MR, Cines DB, Idell S, Neuenschwander PF, Ruppert C, Guenther A, Abraham E, Shetty RS. Induction of tissue factor by urokinase in lung epithelial cells and in the lungs. Am J Respir Crit Care Med 181: 1355–1366, 2010. doi:10.1164/rccm.200901-0015OC. 20194819 PMC2894411

[18] Wong GW, Yasuda S, Madhusudhan MS, Li L, Yang Y, Krilis SA, Sali A, Stevens RL. Human tryptase ε (PRSS22), a new member of the chromosome 16p13.3 family of human serine proteases expressed in airway epithelial cells. J Biol Chem 276: 49169–49182, 2001. doi:10.1074/jbc.M108677200. 11602603

[19] Kaufman RJ. Stress signaling from the lumen of the endoplasmic reticulum: coordination of gene transcriptional and translational controls. Genes Dev 13: 1211–1233, 1999. doi:10.1101/gad.13.10.1211. 10346810

[20] Ribeiro CMP, Boucher RC. Role of endoplasmic reticulum stress in cystic fibrosis-related airway inflammatory responses. Proc Am Thorac Soc 7: 387–394, 2010doi:10.1513/pats.201001-017AW. 21030518 PMC3136959

[21] Rutkowski DT, Kaufman RJ. A trip to the ER: coping with stress. Trends Cell Biol 14: 20–28, 2004. doi:10.1016/j.tcb.2003.11.001. 14729177

[22] Dérand R, Montoni A, Bulteau-Pignoux L, Janet T, Moreau B, Muller JM, Becq F. Activation of VPAC1 receptors by VIP and PACAP-27 in human bronchial epithelial cells induces CFTR-dependent chloride secretion. Br J Pharmacol 141: 698–708, 2004. doi:10.1038/sj.bjp.0705597. 14744818 PMC1574226

[23] Bertrand CA, Zhang R, Pilewski JM, Frizzell RA. SLC26A9 is a constitutively active, CFTR-regulated anion conductance in human bronchial epithelia. J Gen Physiol 133: 421–438, 2009. doi:10.1085/jgp.200810097. 19289574 PMC2664976

[24] Pattison SH, Gibson DS, Johnston E, Peacock S, Rivera K, Tunney MM, Pappin DJ, Elborn JS. Proteomic profile of cystic fibrosis sputum cells in adults chronically infected with Pseudomonas aeruginosa. Eur Respir J 50: 1601569, 2017. doi:10.1183/13993003.01569-2016. 28679606

[25] Pedersen SK, Sloane AJ, Prasad SS, Sebastian LT, Lindner RA, Hsu M, Robinson M, Bye PT, Weinberger RP, Harry JL. An immunoproteomic approach for identification of clinical biomarkers for monitoring disease: application to cystic fibrosis. Mol Cell Proteomics 4: 1052–1060, 2005. doi:10.1074/mcp.M400175-MCP200. 15901828

[26] Griese M, Kappler M, Gaggar A, Hartl D. Inhibition of airway proteases in cystic fibrosis lung disease. Eur Respir J 32: 783–795, 2008. doi:10.1183/09031936.00146807. 18757703

[27] Vermeer PD, Denker J, Estin M, Moninger TO, Keshavjee S, Karp P, Kline JN, Zabner J. MMP9 modulates tight junction integrity and cell viability in human airway epithelia. Am J Physiol Lung Cell Mol Physiol 296: L751–L762, 2009. doi:10.1152/ajplung.90578.2008. 19270179 PMC2681350

[28] Wright C, Pilkington R, Callaghan M, Mcclean S. Activation of MMP-9 by human lung epithelial cells in response to the cystic fibrosis-associated pathogen Burkholderia cenocepacia reduced wound healing in vitro. Am J Physiol Lung Cell Mol Physiol 301: 21743026, 2011. doi:10.1152/ajplung.00226.2010. 21743026

[29] Gaillard EA, Kota P, Gentzsch M, Dokholyan NV, Stutts MJ, Tarran R. Regulation of the epithelial Na^+^ channel and airway surface liquid volume by serine proteases. Pflugers Arch 460: 1–17, 2010. doi:10.1007/s00424-010-0827-z. 20401730 PMC2955882

[30] Tarran R. Regulation of airway surface liquid volume and mucus transport by active ion transport. Proc Am Thorac Soc 1: 42–46, 2004. doi:10.1513/pats.2306014. 16113411

[31] Le Gars M, Descamps D, Roussel D, Saussereau E, Guillot L, Ruffin M, Tabary O, Hong S-S, Boulanger P, Paulais M, Malleret L, Belaaouaj A, Edelman A, Huerre M, Chignard M, Sallenave J-M. Neutrophil elastase degrades cystic fibrosis transmembrane conductance regulator via calpains and disables channel function in vitro and in vivo. Am J Respir Crit Care Med 187: 170–179, 2013. doi:10.1164/rccm.201205-0875OC. 23220915

[32] Prulière-Escabasse V, Clerici C, Vuagniaux G, Coste A, Escudier E, Planès C. Effect of neutrophil elastase and its inhibitor EPI-hNE4 on transepithelial sodium transport across normal and cystic fibrosis human nasal epithelial cells. Respir Res 11: 141, 2010. doi:10.1186/1465-9921-11-141. 20932306 PMC2959028

[33] Birrer P, McElvaney NG, Rüdeberg A, Sommer CW, Liechti-Gallati S, Kraemer R, Hubbard R, Crystal RG. Protease-antiprotease imbalance in the lungs of children with cystic fibrosis. Am J Respir Crit Care Med 150: 207–213, 1994. doi:10.1164/ajrccm.150.1.7912987. 7912987

[34] Sun X, Olivier AK, Liang B, Yi Y, Sui H, Evans TIA, Zhang Y, Zhou W, Tyler SR, Fisher JT, Keiser NW, Liu X, Yan Z, Song Y, Goeken JA, Kinyon JM, Fligg D, Wang X, Xie W, Lynch TJ, Kaminsky PM, Stewart ZA, Pope RM, Frana T, Meyerholz DK, Parekh K, Engelhardt JF. Lung phenotype of juvenile and adult cystic fibrosis transmembrane conductance regulator-knockout ferrets. Am J Respir Cell Mol Biol 50: 502–512, 2014. doi:10.1165/rcmb.2013-0261OC. 24074402 PMC4068938

[35] Butterworth MB, Zhang L, Heidrich EM, Myerburg MM, Thibodeau PH. Activation of the epithelial sodium channel (ENaC) by the alkaline protease from Pseudomonas aeruginosa. J Biol Chem 287: 32556–32565, 2012. doi:10.1074/jbc.M112.369520. 22859302 PMC3463336

[36] Haerteis S, Krappitz M, Bertog M, Krappitz A, Baraznenok V, Henderson I, Lindström E, Murphy JE, Bunnett NW, Korbmacher C. Proteolytic activation of the epithelial sodium channel (ENaC) by the cysteine protease cathepsin-S. Pflugers Arch 464: 353–365, 2012. doi:10.1007/s00424-012-1138-3. 22864553 PMC3448907

[37] Welsh MJ, Ramsey BW, Accurso F, Cutting GR. Cystic fibrosis. In: The Metabolic and Molecular Basis of Inherited Disease (8th ed), edited by Scriver CR, Beaudet AL, Sly WS, Valle D. New York: McGraw-Hill, 2001, p. 5121–5188.

[38] Adkison AM, Raptis SZ, Kelley DG, Pham CTN. Dipeptidyl peptidase I activates neutrophil-derived serine proteases and regulates the development of acute experimental arthritis. J Clin Invest 109: 363–371, 2002. doi:10.1172/jci13462. 11827996 PMC150852

[39] Korkmaz B, Lesner A, Marchand-Adam S, Moss C, Jenne DE. Lung protection by cathepsin C inhibition: a new hope for COVID-19 and ARDS? J Med Chem 63: 13258–13265, 2020. doi:10.1021/acs.jmedchem.0c00776. 32692176

[40] Liu W, Yan M, Liu Y, McLeish KR, Coleman WG Jr, Rodgers GP. Olfactomedin 4 inhibits cathepsin C-mediated protease activities, thereby modulating neutrophil killing of Staphylococcus aureus and Escherichia coli in mice. J Immunol 189: 2460–2467, 2012. doi:10.4049/jimmunol.1103179. 22844115 PMC3424379

[41] Zaidman NA, Panoskaltsis-Mortari A, O'Grady SM. Differentiation of human bronchial epithelial cells: role of hydrocortisone in development of ion transport pathways involved in mucociliary clearance. Am J Physiol Cell Physiol 311: C225–C236, 2016. doi:10.1152/ajpcell.00073.2016. 27306366 PMC5129773

[42] Martino MEB, Olsen JC, Fulcher NB, Wolfgang MC, O'Neal WK, Ribeiro CMP. Airway epithelial inflammation-induced endoplasmic reticulum Ca^2+^ store expansion is mediated by X-box binding protein-1. J Biol Chem 284: 14904–14913, 2009. doi:10.1074/jbc.M809180200. 19321437 PMC2685672

[43] Drumm ML, Ziady AG, Davis PB. Genetic variation and clinical heterogeneity in cystic fibrosis. Annu Rev Pathol 7: 267–282, 2012. doi:10.1146/annurev-pathol-011811-120900. 22017581 PMC4029837

[44] Kunzelmann K, Sun J, Markovich D, König J, Mürle B, Mall M, Schreiber R. Control of ion transport in mammalian airways by protease activated receptors type 2 (PAR‐2). FASEB J 19: 969–970, 2005. doi:10.1096/fj.04-2469fje. 15809358

[45] Sato S, Ito Y, Kondo M, Ohashi T, Ito S, Nakayama S, Shimokata K, Kume H. Ion transport regulated by protease-activated receptor 2 in human airway Calu-3 epithelia. Br J Pharmacol 146: 397–407, 2005. doi:10.1038/SJ.BJP.0706330. 16025139 PMC1576280

